# Editorial: Deciphering microbial metabolites: their impact on gastrointestinal and metabolic health

**DOI:** 10.3389/fnut.2025.1716777

**Published:** 2025-10-20

**Authors:** William J. Massey, Shafiya Imtiaz Rafiqi

**Affiliations:** ^1^Department of Inflammation and Immunity, Cleveland Clinic Research, Cleveland Clinic, Cleveland, OH, United States; ^2^Center for Microbiome and Human Health, Cleveland Clinic Research, Cleveland Clinic, Cleveland, OH, United States; ^3^Department of Medicine, Loyola University Chicago, Chicago, IL, United States

**Keywords:** microbial metabolites, gastroenterology, metabolic disease, microbiota, metabolomics, metabolism

Microbial metabolites are increasingly implicated in human health and disease. Present day research is shifting beyond simply categorizing bacterial taxa in health and disease and attention has shifted toward functional readouts in the form of metabolites. Understanding the mechanisms by which these metabolites affect health and disease will ultimately lead to novel approaches for preventing, treating, and curing disease (1). At present, however, there is a large gap in understanding the mechanisms that microbial metabolites act upon in homeostatic and diseased contexts. Toward the goal of filling this gap in knowledge we present this Research Topic, which assembles 13 articles, including four review and nine original research papers, that collectively highlight the translational promise of microbial metabolites, covering the role of microbial metabolites in typical gastrointestinal (GI) and metabolic diseases, but also diseases of other gut-organ axes.

In the realm of diseases falling under the category of gastroenterology, Liu Y. et al. characterized the metabolite profile of 10 colorectal cancer (CRC)-associated bacteria and identified many amino acid and nucleotide metabolites traceable to specific CRC-associated microbes providing a basis for mechanistic studies to understand how these metabolites correlate with or affect disease outcomes and phenotypes. This is particularly important in context of understanding tumor microbiome interactions, highlighting the potential for microbial metabolites to directly shape oncogenic microenvironments. At the other end of the GI tract, bile is released into the duodenum from the common bile duct, which can become occluded by gallstones formed from bile acids. Tan et al. reviewed what is known about the role of the gut microbiome in gallstone formation in which metabolites such as short chain fatty acids (SCFAs), trimethylamine-N-oxide (TMAO), and secondary bile acids (2°BAs) are altered. With respect to therapeutic opportunities, the authors propose multiple modes of targeting the microbiome, including direct supplementation with metabolites such as tauroursodeoxycholic acid and butyrate. In an investigation of the gut-liver axis, Shi et al. investigated the role of the microbiome in hepatitis B viral-related cirrhosis using prospectively collected fecal samples. Through metabolomics, they identified lipid metabolism as a dysregulated pathway in viral cirrhosis patients. Specifically, they observed reduced tocopherols, 21-hydroxy-pregnenolone, and 5β-coprostanol as well as elevated homoserine, fucose, N-acetyl-D-glucosamine, and p-hydroxymandelic acid in cirrhosis patients vs. healthy controls. These findings put forth fecal biomarkers for identification of early cirrhosis and suggest that either dysregulation of lipid metabolism and uptake alters the microbiome or vice versa in viral cirrhosis. Xiaohua et al. sought to understand the role of probiotics in the management of pediatric food allergy by conducting a systematic review and meta-analysis using outcomes including Scoring Atopic Dermatitis (SCORAD), IgE levels, and quality of life scores. From their analyses, the authors find that *Lactobacillus GG* is most effective probiotic across 16 randomized clinical trials in reducing SCORAD scores and increasing quality of life. *Lactobacillus acidophilus* showed the strongest effect on IgE levels. These analyses prompt further studies regarding the molecular mechanisms, and perhaps metabolites, by which these microbes may protect against food allergy. Zhou et al. investigated the role of the Sishen pill (SSP), a traditional Chinese medicine formula for its therapeutic effect on diarrhea. By administering adenine and *Foliuem sennae* (senna leaf), the authors induced diarrhea in mice and treated half of these animals with SSP and allowed the other half to recover spontaneously. While the only metabolic product measured in the study was uric acid, whose level rose in mice administered adenine and *F. sennae* vs. controls and was normalized by treatment with SSP, the authors measured intestinal enzyme and microbial activities using various biochemical assays. Through these results they show clear metabolic effects of SSP in normalizing the changes induced by the diarrhea model; however, further work is needed to know if these changes are due to microbial metabolism or microbe induced changes to host metabolism.

In addition to research on GI diseases, the Research Topic includes six manuscripts covering metabolic diseases. The first manuscript by Liu T. et al. showed that a *Zingiber striolatum* extract (ZSE) alleviates type 2 diabetes mellitus (T2DM) in an interventional model comparable to the effects of metformin. Interestingly, the ZSE, but not metformin restored the *Firmicutes/ Bacteroides* ratio to level the non-T2DM control group, although both ZSE and metformin increased the production of all measured SCFAs. On SCFAs, Miyamoto et al. investigated SCFA production by the probiotic species, *Acidipropionibacterium acidipropionici* and signaling through one of the known G-proteins coupled receptors (GPCRs) for SCFAs, GPR41. *A. acidipropionici* treatment resulted in increased plasma acetate and butyrate as well as cecum acetate and propionate in mice. Coincident with these changes in SCFA production, wild type mice fed a high fat diet developed less glucose intolerance and insulin resistance when treated with *A. acidipropionici*, however, these metabolic effects were not observed in GPR41 knockout mice. Chen et al. investigated another microbe-GPCR axis in zebrafish. In this study, it was shown that *Lactobacillus fermentum* E15 reduced blood and liver lipids in high cholesterol diet fed zebrafish, which reduced lipid synthetic gene expression. The mechanism of these effects was shown through loss-of-function studies to be isovaleric acid acting on GPR43. In an epidemiological study, Cai et al. used data from the National Health and Nutrition Examination Survey (NHANES) to study the correlation between hyperuricemia and the dietary index for gut microbiota (DI-GM), which is quantified based on 14 foods or nutrients that were classified as beneficial or unfavorable components. Each component was scored 0 or 1 based on sex-specific median intakes, and scores were summed to develop the overall DI-GM score (2). In alignment with studies of other chronic diseases and their relationship to DI-GM, Cai et al., found that high DI-GM is inversely correlated with the risk of hyperuricemia, prompting further mechanistic studies investigating how dietary factors may affect microbial metabolite production to influence the development of hyperuricemia. Ma et al. reviewed the effects of acupuncture on the so-called microbiota–gut–brain axis (MGBA), with reference to the effects of nutrition on obesity via the MGBA. The authors suggest numerous parallels between acupuncture and nutritional interventions in obesity, including effects on SCFAs and their receptors, GLP-1 secretion, microbial community structure, and gut barrier function. These insights suggest that acupuncture may serve to complement or synergize with traditional nutrition-based interventions in obesity through modulation of the gut microbiome and its metabolites. These contributions are valuable because they broaden the conceptual scope of the research and encourage a necessary link between traditional approaches and modern biomedical science. Lastly, Bahitham et al. offered a systematic review of microbial metabolites in metabolic dysfunction associated steatotic liver disease (MASLD) and metabolic dysfunction associated steatohepatitis (MASH). From their review of human and animal studies, they find that microbial taxa within specific regions of the gut and metabolites have great potential for diagnosis and therapies in MASLD/MASH, while highlighting the need for longitudinal studies with diverse cohorts that leverage multi- omics technologies.

Excitingly, in addition to the work submitted to this Research Topic on classical GI and metabolic diseases, there was additional work that highlighted the potential for the gut microbiome in regulating granulomatous lobular mastitis (GLM) and female infertility, which highlight the little studied gut-breast and gut-reproductive axes. Specifically, GLM is characterized by benign inflammation of the breast that most commonly occurs in women 20–40 years of age (3) and is typically managed by antibiotics, corticosteroids, immunosuppressive agents, or surgery (4). Dai et al. sought to expand upon existing work showing that the gut microbiome may be involved in GLM by measuring SCFAs in addition to performing 16s rRNA sequencing, which confirmed the findings of the previous studies. When examining short-chain fatty acids (SCFAs) in the GLM stool samples, the authors observed an increase in butanoic acid compared to control subjects. More critically, the levels of isohexanoic acid were found significantly higher in patients who went on to have recurrent disease. Cheng et al., like the study by Cai et al. mentioned above, used the DI-GM to connect diet and gut microbiome interactions to disease outcome with regards to female infertility. The authors found a significant negative association was observed between DI-GM score and female infertility, suggesting that dietary intervention with the goal of altering the gut microbiota and its metabolites may lead to reduced female infertility.

This research area has emerged at a particularly significant juncture. As rates of GI and metabolic diseases climb worldwide, and as diets continue to shift globally, the modulation of microbial metabolites presents a promising non-invasive, readily modifiable approach for both prevention and therapeutic intervention. However, substantial challenges remain including reproducibility issues, and the rigorous clinical validation required to translate metabolite signatures into validated diagnostic tools. To overcome these hurdles, we advocate for the research community to prioritize interdisciplinary collaboration, bringing together clinicians, data scientists, and researchers. Finally, the editorial team of the Research Topic would like to thank all the authors, reviewers, and readers who have contributed to and engaged with this collection. With all your support, we hope this Research Topic may highlight the important and growing field of microbial metabolites while providing inspiration for novel future studies in this exciting research field that someday may be an integral part of patient care.
